# Is the Brazilian pharmaceutical policy ensuring population access to essential medicines?

**DOI:** 10.1186/1744-8603-8-6

**Published:** 2012-03-21

**Authors:** Andréa Dâmaso Bertoldi, Ana Paula Helfer, Aline L Camargo, Noêmia U L Tavares, Panos Kanavos

**Affiliations:** 1 Postgraduate Program in Epidemiology, Federal University of Pelotas, Rua Marechal Deodoro 1160, 96020-220, Pelotas, Brazil; 2 London School of Economics and Political Science, Houghton Street, London, WC2A 2AE, UK; 3 Universidade do Vale do Rio dos Sinos, Av. Unisinos 950, 93022-000, São Leopoldo, Brazil; 4 Porto Alegre Federal University of Health Sciences, Rua Sarmento Leite 245, 90050-170, Porto Alegre, Brazil; 5 Universidade da Região da Campanha, Rua Brigadeiro Mercio 72, 96400-720, Bagé, Brazil

**Keywords:** Health expenditures, Economics, Pharmaceutical, Drugs, Generics, Developing countries

## Abstract

**Background:**

To evaluate medicine prices, availability and affordability in Brazil, considering the differences across three types of medicines (originator brands, generics and similar medicines) and different types of facilities (private pharmacies, public sector pharmacies and “popular pharmacies”).

**Methods:**

Data on prices and availability of 50 medicines were collected in 56 pharmacies across six cities in Southern Brazil using the World Health Organization / Health Action International methodology. Median prices obtained were divided by international reference prices to derive the median price ratio (MPR).

**Results:**

In the private sector, prices were 8.6 MPR for similar medicines, 11.3 MRP for generics and 18.7 MRP for originator brands, respectively. Mean availability was 65%, 74% and 48% for originator brands, generics and similar medicines, respectively. In the public sector, mean availability of similar medicines was 2–7 times higher than that of generics. Mean overall availability in the public sector ranged from 68.8% to 81.7%. In “popular pharmacies”, mean availability was greater than 90% in all cities.

**Conclusions:**

Availability of medicines in the public sector does not meet the challenge of supplying essential medicines to the entire population, as stated in the Brazilian constitution. This has unavoidable repercussions for affordability, particularly amongst the lower socio-economic strata.

## Background

The Brazilian Universal Health System (SUS) is committed to offer high-quality health care to the entire population, including the distribution free of charge of a list of essential medicines aimed at treating the most prevalent diseases in the population. Also, the government provides expensive medicines for treating rare diseases or medicines targeting small groups (e.g. Crohn’s disease, hepatitis B and C) free of charge, based on clinical protocols and therapeutic guidelines from the Ministry of Health [1]. Charging patients for medicines is strictly prohibited in the public system. In real life, however, medicines are often not available when needed. Studies carried out in Brazil have shown that, on average, 40% of the medicines prescribed in public primary health care were not available when needed [2,3]. Although access to medicines in Brazil is high [4,5], socioeconomic inequities are observed [4]. Despite poor families receiving more medicines free of charge from government-funded sources than the better-off, 25.5% of the medicines obtained by the bottom income quintile of the population are paid for out-of-pocket [4].

Overall, Brazilian families spend 9% of their household income on health, and medicines account for the largest proportion of all health expenses [6]; 31.5% of monthly health expenses and 2% of monthly family income were shown to be spent on medicines[7]. Although the majority of the Brazilian population uses SUS, 25% of all families pay for private health insurance [8], which in Brazil does not cover the costs of medicines used in ambulatory care. In 2007, Brazilian families spent 10 times more money on medicines than the government [9].

In order to compensate for the limitations in the availability of free medicines in the public sector, the Brazilian government launched the “popular pharmacy” programme in 2004. The programme sells medicines at low prices to the population, particularly those who use private health facilities but who have difficulty in buying their medicines in private pharmacies [10]. There are two types of “popular pharmacies”: (a) those which are run by the state, city governments, universities or other health-related institutions. In these pharmacies, medicines from a list comprising 95 molecules selected on the basis of the most prevalent health problems in Brazil or which are expensive for individuals to acquire are sold at cost prices. From here onwards we refer to these facilities as exclusive “popular pharmacies”; (b) those which are run in partnership with private pharmacies using a system of co-payments. This category was created in 2006 as a means to expand the popular pharmacy program. In these facilities the government covers 90% of the price whereas the patient pays the remaining 10%. However, only a list of anti-hypertensive, anti-diabetic and contraceptive medicines are sold in this way [10].

There are three types of medicines available in the Brazilian market: originator brands, generics and similar medicines [11]. All generic medicines must be commercialized with no brand. Generics are medicines which are interchangeable with the originator brand (subject to the standard bioequivalence and bioavailability tests) [12]. Bioequivalence is a term in pharmacokinetics used to assess the expected in vivo biological equivalence of two proprietary preparations of a drug. If two products are said to be bioequivalent it means that they would be expected to be, for all intents and purposes, the same. Birkett (2003) defined bioequivalence by stating that, *"two pharmaceutical products are bioequivalent if they are pharmaceutically equivalent and their bioavailabilities (rate and extent of availability) after administration in the same molar dose are similar to such a degree that their effects, with respect to both efficacy and safety, can be expected to be essentially the same. Pharmaceutical equivalence implies the same amount of the same active substance(s), in the same dosage form, for the same route of administration and meeting the same or comparable standards*”[13].

Similar medicines are all the others available on the market. This type of medicine is comparable to “branded generics” [14] described in the international literature. Branded generics are generics whose manufacturers launch them with a particular brand name, which can be a ‘fantasy’ or invented name (protected by trademark law), or the name of the manufacturer followed by the name of the molecule [14]. Until recently, similar medicines were required to undergo pharmaceutical equivalence, but not bioavailability tests, making them frequently cheaper than generics. However, there is a transition period expiring at the end of 2014 enabling them to be on the market without these tests [15].

Medicine access relates both to affordability and availability (available stock of essential medicines in pharmacies). Availability, particularly in the public sector, is an issue of considerable concern in Brazil. Recent research found that for 71% of medicines the availability of generics was below 10% [16]. International evidence, using data from 36 low and middle-income countries, showed that in the public sector, availability ranged from 29 to 54% and prices for private patients were 9 to 25 times higher than international reference prices for generics and 20 times higher for originator products [17].

Retail prices are the result of a series of factors, such as procurement prices. In the private sector, the volume of purchases in the pharmacies is usually low, resulting in purchases from wholesalers, increasing the price for consumers [18]. There is an exception related to the big chains of pharmacies, which can centralize the procurement process decreasing significantly the medicine costs, usually transferring it to the final consumer. The Brazilian retail market is formed mainly by independent pharmacies (around 90% of the pharmacies). The five main chains of pharmacies represent only 2.8% of the total number of pharmacies in the country, although they were responsible for 23% of the market share of medicines in 2010 [19].

In the public sector, a series of different procurement models is used by governments from developing countries to purchase medicines and other health products. An increasing number of countries have opted to decentralize the procurement process as an effort to answer to local needs. The decentralized purchase involves different levels of responsibility in the procurement process at the federal, state and city levels [20].

In Brazil, the procurement of medicines in the public sector is decentralized at different government levels. All purchases are carried out by invitation, following a Federal Law [21], and usually are made directly from the manufacturers. Considering the heterogeneity of the Brazilian municipalities, regarding size as well as organization, there are relevant differences in the procurement prices of medicines [22]. However, many Brazilian municipalities are already organized to purchase in large scale, which may lead to better prices [23].

Taking into account that both the availability of medicines, particularly in the public sector, and their price in the private sector are important determinants of access to medicines, this study aims to investigate medicine prices, availability and affordability in the Brazilian state of Rio Grande do Sul, located in the South of the country. Particular attention is given to the three types of medicines available in Brazil, notably originator brands, generics and similar medicines and the different types of facilities (private pharmacies, public sector pharmacies and “popular pharmacies”).

## Methods

The paper uses the World Health Organization / Health Action International (WHO/HAI) methodology [24] to study price levels, availability and affordability of medicines in the Southern Region of Brazil. This methodology enables researchers to investigate the (a) prices people pay for key medicines^a^; (b) variability of prices and availability of medicines in different market segments (public, private and other medicine outlets); (c) differences in prices and availability between originator brands and generics; and (d) affordability of medicines among “ordinary”^b^ people [24].

The methodology relies on conducting surveys, whose key design elements are: (a) data collection takes place in six areas of a selected country or state (in the case of large countries, like Brazil); (b) the survey includes pharmacy outlets from both the public and private sectors; (c) up to 50 medicines are surveyed; (d) data on prices and availability of medicines are obtained by data collectors during visits to the selected pharmacy outlets; (e) for each medicine and pharmacy outlet, data are collected on the originator brand and the lowest-priced generic.

The current study has drawn data from the southern region of Brazil (“South”), which is one of five geographical regions of the country (South, Southeast, Midwest, Northeast and North). The South Region consists of three states (Parana, Santa Catarina and Rio Grande do Sul). The sample used in this study was drawn from the state of Rio Grande do Sul, which has a total population of 10.5 million, representing 5.7% of the country’s total population [25].

Data were collected from six cities within the state: (a) the state capital (Porto Alegre, 1.4 million inhabitants); (b) two cities in the Southern part of the state (Pelotas and Bagé), which are the poorest in the state; (c) one city in the metropolitan area of the state capital (São Leopoldo); (d) one city in the richest part of the state (Caxias do Sul); and (e) one city in the central region of the state (Santa Cruz do Sul). The total population of these six cities represents one quarter of the state’s total population.

The state of Rio Grande do Sul ranks 6^th^ in the country (out of 26 states) in terms of gross domestic product (GDP), with a GDP per capita above the national average of R$13,720 (US$5,900) per capita. Poverty and income inequality are below the national average [26]. GDP varies considerably across the six study cities, with three having a GDP per capita below the national and state average (São Leopoldo, Pelotas and Bagé) while the remaining three (Porto Alegre, Santa Cruz do Sul e Caxias do Sul) are above these averages [26]. The proportion of individuals below the poverty threshold was under 30% in all cities (below the national average of 36.5%) [25].

The sampling for the survey reflected all ambulatory types of pharmacy outlets. In each of the six cities, four public sector facilities with pharmacies were randomly selected from a list of all public sector facilities that dispense medicines in each city. The only exception was São Leopoldo, where only two health facilities dispensed medicines (n = 22). Five private pharmacies per city were also selected and were matched to the public sector facilities based on their proximity to them (n = 30). In addition, all exclusive “popular pharmacies” (n = 4) were included in the study. The total sample size was N = 56. Pharmacies were visited only once, and interviewers requested to see the packaging of all medicines surveyed. All pharmacies agreed to take part in the study and a written informed consent form was signed prior to data collection.

Data were collected from the beginning of November 2008 to the end of January 2009. Prices and availability of 50 medicines were investigated. Of these, 29 medicines were part of the WHO/HAI global and regional core lists, whereas the remainder, the supplementary list, were selected from the national (RENAME) and municipal (REMUME) lists of essential medicines [27]. Medicines from the global core list are to be included in all medicine price surveys, in order to enable international comparisons. The regional core list is study-specific and accounts for regional differences in medicine usage, but still allows for cross-country comparisons within the same broad geographical region. The supplementary list of 21 medicines is selected at the country level considering local particularities. The REMUME is part of the RENAME list, following the local epidemiological profile and is obtained directly from the health secretariats of each city. The 50 medicines selected correspond to 12.6% of the 342 medicines that were part of the national essential list (RENAME) in 2008. The names of all medicines included in each list (global, regional and supplementary list) are presented in Additional file 1: Table S1.

Antidepressants, anti-epileptics and anxiolytics are subject to controlled dispensing and are distributed by a restricted number of pharmacies. Due care was exercised to include as many of these pharmacies in the sample as possible. For each selected medicine, data for the following variables were obtained: availability at each sampled outlet, patient price for the originator brand, the lowest-priced generic and the lowest-priced similar medicine.

The study endpoints comprised three measures: availability, medicine prices and affordability. Availability was defined as the proportion of pharmacies in which the medicines were available at the time of the survey. The mean availability percentage was calculated as the average percentage value from all medicines. Prices were presented as median price ratios (MPR). The MPR is the ratio of a medicine’s median price across outlets divided by the Management Science for Health (MSH) median international reference price for the year preceding the survey (2007) [28]. This study specifically looks at retail prices in both private and exclusive “popular pharmacies”. Prices collected in the public sector were procurement prices and they were not analyzed.

Affordability was estimated as the number of days that the lowest-paid unskilled government worker earning the minimum monthly wage would need to work in order to purchase a complete course of treatment in a private pharmacy. The gross minimum monthly wage in Brazil in the end of 2008 was R$415 (US$178.50); after excluding 8% for national insurance contributions, the adjusted value was R$381.80 (US$165) [29].

Data entry and analyses were performed using the computerized Excel^R^ WHO/HAI Medicine Pricing Workbook, enhanced for the purpose of including originator brands, generics and similar medicines, thus taking into account the peculiarities of the Brazilian context^c^.

MPRs in private pharmacies were only calculated if the medicine was available on, at least, four facilities. In the case of “popular pharmacies”, due to the small sample size, calculations of MPR were performed if the medicine was available in at least one facility. Median price differences across the three types of medicines included only medicines for which the pair was found in at least one facility.

In order to estimate the mean availability of medicines in the exclusive “popular pharmacies”, only 36 medicines were included in this part of the analysis, as the remaining 14 were not commercially available in this type of facility. To avoid underestimation of availability of generic medicines, all cases in which generics were not available in the Brazilian market were excluded from the calculations of availability.

The project was approved by the Ethics Committee from the Municipal Secretariat of Porto Alegre. People responsible for providing information on each pharmacy provided written informed consent.

## Results

Additional file 1: Table S1. describes all medicines studied in terms of pharmacological groups, their presence or not in the Brazilian list of essential medicines (RENAME) and availability of the three types of medicines in the public and private sectors. Out of the 50 medicines studied, 43 are part of RENAME. The following medicines, which come either from the global or from the regional list, are neither part of RENAME nor REMUME: atorvastatin 10 mg tab, clotrimazole 10 mg/g cr, ibuprofen 400 mg cap and simvastatin 20 mg tab.

Table 1 shows the mean proportion of availability in the public sector. As expected, no originator brands were found in public sector facilities. Mean availability of similar medicines was 2–7 times greater than that of generics; this difference was larger in the poorest cities (São Leopoldo, Pelotas and Bagé) compared to the wealthier ones. Availability, independently of the type of medicine (generic or similar), was 78.3% in Porto Alegre, 71.5% in Santa Cruz, 77.0% in Caxias do Sul, 80.3% in São Leopoldo, 68.8% in Pelotas and 81.7% in Bagé.

**Table 1:** Mean availability (%) of medicines^1^ in the public sector (n = 22 facilities)

**City**	**N**	**Lowest-priced generic**		**Lowest-priced similars**	
		**Mean %**^**2**^	**SD %**^**3**^	**Mean%**	**SD%**
Porto Alegre	30	23.3	41.9	55.8	41.9
Santa Cruz do Sul	38	14.5	31.6	59.9	39.7
Caxias do Sul	35	17.1	32.5	62,9	39.5
São Leopoldo	33	13.6	31.3	69.7	43.2
Pelotas	32	8.9	21.0	61.7	39.6
Bagé	26	11.5	27.6	70.2	38.1

^1^Only medicines included in the list of essential medicines within each city were included in this analysis.

^2^Mean avalability %^3^Standard deviation %.

Sample of six cities from the Rio Grande do Sul state, Brazil, 2008-9.

Mean availability of lowest-priced generics was lowest in “popular pharmacies” of the two poorest cities (São Leopoldo e Bagé) and availability of lowest-priced similar medicines was lower in the two wealthier ones (Porto Alegre e Caxias do Sul) (Table 2). No originator brands were found in “popular pharmacies”. Mean availability, independently of the type of medicine (generic or similar), was greater than 90% in all cities: 91.6% in Porto Alegre, 91.6% in Caxias do Sul, 97.2% in São Leopoldo and 97.3% in Bagé. In relation to prices, the values were very similar for the lowest-priced similar medicines, but ranged from 2.6 to 4.1 MPR for the lowest-priced generics.

**Table 2:** Mean availability (%) and price of the 36 medicines dispensed by the Popular Pharmacy (n = 4 facilities)

**City**	**Lowest-priced generic**			**Lowest-priced similar**		
	**n**^**1**^	**Price**	**Availability**	**n**	**Price**	**Availability**
		**MPR**^**2**^**(Min—Max)**	**Mean % (SD)**		**MPR (Min—Max)**	**Mean % (SD)**
Porto Alegre	8	3.63 (1.30–11.41)	22.2 (42.2)	25	2.61 (0.33–15.17)	69.4 (46.7)
Caxias do Sul	8	2.88 (1.30–11.41)	22.2 (42.2)	25	2.61 (0.33–15.17)	69.4 (46.7)
São Leopoldo	7	2.64 (1.30–11.41)	19.4 (40.1)	28	2.86 (0.33–15.17)	77.8 (42.2)
Bagé	6	4.12 (1.30–11.41)	16.7 (37.8)	29	2.61 (0.33–15.17)	80.6 (40.1)
Total	10	2.88 (1.30–11.41)	20.1 (35.8)	31	2.64 (0.33–15.17)	74.3 (38.0)

^1^ Number of medicines included in the analysis of the MPR.

^2^*MPR* median price ratios (= median prices / IRP) → *IRP* international reference prices (MSH, 2007).

Sample of six cities from the Rio Grande do Sul state, Brazil, 2008-9.

Table 3 presents data on the availability of medicines and prices in the private sector. Mean availability of originator brands ranged from 48 to 91%; equivalent figures were 63 to 88% for lowest-priced generics and 39 to 55% for lowest-priced similar medicines. The availability of originator brands was higher in the two poorest cities (Pelotas and Bagé). Overall, mean availability was 65%, 74% and 48% for originator brands, generics and similar medicines, respectively. In terms of MPR, values ranged from 8.6 for lowest-priced similar medicines to 18.7 for originator brands; MPR was 11.3 for lowest-priced generics.

**Table 3:** Mean availability (%) of medicines and prices in the private sector (n = 30 pharmacies)

**City**	**Originator brand**			**Lowest-priced generic**			**Lowest-priced similar**		
	**n**^**1**^	**MPR**^**2**^**(min—max)**	**Availability (SD)**^**3**^	**n**	**MPR (min—max)**	**Availability (SD)**	**n**	**MPR (min—max)**	**Availability (SD)**
Porto Alegre	23	17.06 (2.70–94.58)	57.6% (37.6%)	21	9.97 (0.93–34.36)	63.3% (28.6%)	18	15.61 (3.17–37.87)	54.4% (33.1%)
Santa Cruz do Sul	18	25.99 (2.60–87.57)	57.6% (30.1%)	11	9.88 (0.85–37.76)	68.8% (22.8%)	23	18.34 (1.88–32.49)	39.2% (32.8%)
Caxias do Sul	17	30.63 (2.70–94.68)	58.0% (23.3%)	25	11.33 (0.93–48.92)	71.2% (25.6%)	14	12.40 (1.86–37.48)	47.2% (30.4%)
São Leopoldo	15	29.40 (4.90–66.45)	48.4% (28.3%)	17	8.42 (0.85–33.93)	65.6% (26.3%)	17	8.18 (2.75–38.82)	52.0% (31.6%)
Pelotas	35	19.08 (2.70–107.47)	75.6% (26.9%)	34	9.76 (0.87–48.95)	85.6% (25.3%)	10	9.36 (2.94–22.37)	41.2% (27.5%)
Bagé	46	16.73 (1.32–162.53)	91.0% (15.7%)	39	9.55 (0.36–49.82)	88.4% (20.6%)	20	8.81 (1.26–36.45)	54.8% (33.3%)
All	49	18.66 (1.36–168.41)	64.8% (22.8%)	42	11.32 (0.84–54.94)	73.8% (18.8%)	45	8.60 (1.11–38.84)	48.1% (26.8%)

^1^ N = Total number of medicines included in the analysis. To calculate the MPR, we only included medicines which were available on at least four facilities.

^2^*MPR* median price ratios (= median prices / IRP) → *IRP* international reference prices (MSH, 2007).

^3^ Mean avalability % (standard deviation).

Sample of six cities from the Rio Grande do Sul state, Brazil, 2008-9.

**Figure 1:**
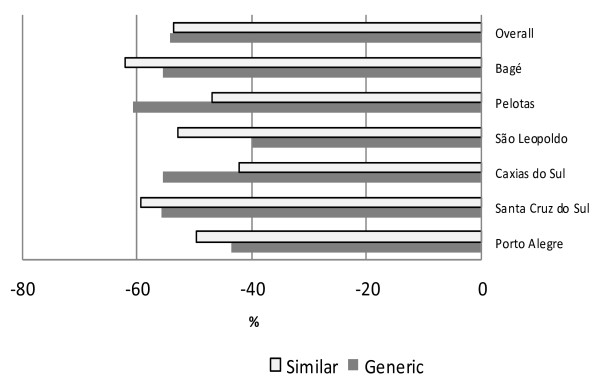
**Median price differences for matched pairs of medicines.** Median price difference (%) of generic and similar medicines in relation to originator brand medicines in the private sector for matched pairs of medicines available on at least one facility. Sample of six cities from the Rio Grande do Sul state, Brazil, 2008–9.

Taking into account all cities together, the price of both generics and similar medicines is roughly half the price of originator brands (Figure 1). The greatest difference between similar medicines and originator brands was found in Bagé (similar medicines were 62% cheaper than originator brands) whereas the smallest difference was found in São Leopoldo (generics were 40% cheaper than originator brands).

**Figure 2:**
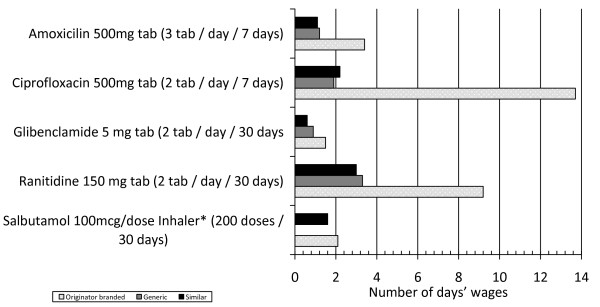
**Affordability of selected medicines.** Affordability of generic, originator brand and similar medicines using a list of selected medicines. Sample of six cities from the Rio Grande do Sul state, Brazil, 2008–9. * Generic medicines of the salbutamol 0.1 mg/dose inhaler were not available at the time of data collection.

The price difference recorded between originator brand and generics or similar medicines have significant implications for affordability (Figure 2). A seven-day treatment with originator brand ciprofloxacin 500 mg would cost 13.7 days of salary; the equivalent figures were 2.2 and 1.9 days for similar and generic medicines. For a seven-day treatment with amoxicillin 500 mg, the values are 3.4 (originator brand) and 1.1 and 1.2 days (similar and generic medicines). For a 30-day diabetes treatment with glibenclamide 5 mg, the differences were very small (values around 1.0 day for similar and generic medicines and 1.5 for originator brand). A 30-day ulcer treatment with ranitidine 150 mg would cost 9.2 (origi-nator brand) and 3.3 and 3.0 days (generics and similar medicines respectively). The originator medicine for a 30-day treatment for asthma (salbutamol inhaler, 200 doses) would cost 2.1 (originator brand) and 1.6 days (similar medicine). No generics are available for salbutamol inhaler in the Brazilian market.

## Discussion

Our results suggest, first, that prices of generics and similar medicines are, on average, half those of originator brands. The MPR for all medicines (brands, generics and similars) was found in all cases to be significantly greater than one, indicating that prices in Brazil are higher than international reference prices. Public sector availability of generics or similar medicines is lower than expected and, consequently, patients resort more often to purchasing medicines in private pharmacies, where availability is higher, but prices are high and patients have to pay fully out-of-pocket, thus impacting affordability.

In private sector pharmacies, generics were the most likely medicines to be available, followed by originator brands and similar medicines. Because reimbursement of medicines is very rare in Brazil, private pharmacies typically try to offer all types of medicines, so that consumers from all social classes are able to obtain their medicines.

In the recent past (beginning of the generic’s implementation in Brazil), generics accounted for only a small proportion of the medicines market in Brazil [30], because people were more likely to buy either the cheapest medicine (usually a similar, at that time) or the originator brand based on the belief that the latter were of better quality than generics or similar medicines. In recent years, the market share of generics has increased significantly both in absolute terms and in comparison with originator brands and similar medicines [31]. Because of the increased number of generics in the market and the change in the similar’s regulation about quality tests and product registration, we can find similars which are cheaper or more expensive than generics, depending on the manufacturer and whether or not they were already adjusted to the new regulation.

Mean availability of generics in the private sector was 73.8%, a figure comparable to those observed in the Americas, Eastern Mediterranean, Europe and Southeast Asia and higher than those in Africa or Western Pacific, whereas mean availability of originator brands (64.8%) was similar to that found in upper middle income countries (61.8%) [17]. Private sector MPRs were lowest for the similar medicines, a finding consistent with previous research [16].

Availability of medicines in the public sector was at or above 69% in all cities, and was slightly lower than availability investigated in another study [32]. Placing our availability findings in an international context we find that availability in Brazil is lower than that of Sudan (82%) [33], but higher than that in Malaysia (5-40%, depending on the medicine) [34], India (0-30%, depending on the region of the country) [35] and Chinese rural areas (38.9%) [36].

Still, our findings on availability are poor considering that all Brazilian citizens should have access to all essential medicines free of charge, a right which is constitutionally protected [37]. The lack of several medicines in the public sector forces patients to purchase their medicines out-of-pocket in private sector outlets and may lead to catastrophic health expenses [38] and/or undertreatment [3].

Our affordability findings varied by therapeutic class and type of medicine and are attributable to poor availability in the public sector. Although the lowest-paid unskilled government worker salary was used as a measure of affordability it is likely that a significant part of the population earns less [17]. This is a limitation, considering that this method cannot capture the wage for the average worker. However, the WHO/HAI method to evaluate affordability has the advantage of being easily applicable and, consequently, used in many places around the world allowing international comparisons. In order to deal with this limitation, other measures have been applied, e.g., using an impoverishment method as a metric of affordability. Using this approach, purchases can be estimated by determining pre- and post-payment incomes [39]. In Brazil, 32.8% of the population live below the poverty line of US$1 per day [25]. For these people, any out-of-pocket expense related to medicines could be catastrophic.

Availability of generic medicines in the public sector was 9–23% in all cities studied, whilst availability of similar medicines was 56 – 70%. These are comparable to those in other regions of the country, where similar medicines are more widely available (86.4%) than generics (25%) [16].

The different levels of availability observed in each city may partly reflect two trends; first, decentralization of the Brazilian public health system, whereby municipalities fund a proportion of medicines directly out of their budgets; and second, differences in medicines distribution, where supply of public sector facilities is often problematic (inadequate quantities, problems with frequency and time of distribution, and inefficient stock control, among others) [32,40].

These supply problems have been reported in some states of Brazil. A study carried in Minas Gerais state indicated that in 53% of the distribution centers studied, there was disagreement between physical count and corresponding records. In addition, 17% of the municipalities were not working based on a list of essential medicines [41]. Another study detected the lack of standard operational procedures to medicine’s stock, no access restrictions to non-authorized personnel, lack of training to those working in the medicine’s stock and lack of technical criteria to select the medicines to be available to the population [42]. Also in Rio Grande do Sul state, a study including 20 municipalities detected problems as: (a) absence of the pharmacist in 75% of the sample; (b) 22% of the essential items of good storage practices were not met; (c) 10% of the municipalities did not have stock control procedures [43].

Aware of these problems, the Department of Pharmaceutical Care of the Brazilian Ministry of Health has started to propose alternatives to improve the situation. In partnership with Brazilian universities, it has been offering courses to pharmacists working in public health services. It has also developed a software to manage the pharmaceutical care in the public health system. The tool allows inventory control, traceability of drugs distributed and dispensed, a profile of consumption and monitoring of medication use, among other features [44].

The availability of medicines which are part of the list of the “popular pharmacies” was high (>90% in all cities). Similar results have been described in other regions of the country [45]. As in public sector facilities, similar medicines are also more frequently available than generics in “popular pharmacies”. Retail prices in “popular pharmacies” are standardized, and, therefore, differences observed across cities reflect the fact that different medicines were available in each facility, thus resulting in a different mean value. “Popular pharmacies”, in theory, may represent an important additional source of access to medicines for the population, given that prices are much lower than those observed in private pharmacies. Yet, the number of “popular pharmacies” is low and the list of medicines provided is limited. The fact that a high proportion of the users of the programme are also SUS users – 39.2% according to a recent study [46] - suggests that the lack of medicines in the public system is a reality, and patients often need to use “popular pharmacies” in order to obtain essential medicines, which should be supplied free by the government.

The highest median price difference between originator brand and the lowest-priced generic was 60%. Between 2000 to 2004, when generic medicines were launched in Brazil, the mean price difference between generics and originator brand products was 40% but this tended to increase over time [47]. Both types of medicines presented absolute increases in their prices, but the increase in the price of the originator brand was relatively higher than that of the generic equivalent(s) [47].

The government department which regulates the market and establishes the criteria for medicine price adjustments is the *Câmara de Regulação do Mercado de Medicamentos (CMED)*. The objective of this department is to encourage the availability of medicines and competition in the sector [48]. The current regulation defines the criteria for the annual readjustment of prices defined by the level of market competition based on the market share of generics. The regulation also establishes that the manufacturers of medicines may readjust the price of their products following an index fixed in three different bands. In order to adjust prices, manufacturers are required to submit a report on market activity to CMED. Only phytotherapic, homeopathic and other medicines which can be sold with no control of prices are not subject to the model of the top price adjustment [49].

CMED also establishes that in any sale carried out by medicine manufacturers or wholesalers targeted to the public or private sectors, there is a ceiling on the manufacturer price. In addition there is a maximum sale price to the government. In this case, a percentage of the price should be discounted from the manufacturer’s price in sales targeted to the government. There is also a ma-ximum sale price in pharmacies and drugstores for consumers. These prices include taxes [18].

According to the *Agência Nacional de Vigilância Sanitária* (ANVISA), which plays the role of executive secretary for CMED, after the launch of CMED, medicines costs started to decrease and actions for the regulation of prices interrupted the increase trend for the costs of these products in the country. Despite this fact, prices in Brazil could be less expensive if taxes were lower. On average, 36% of the price paid for medicines in the country goes to the government due to taxes along the supply chain. One of the main problems for the pharmaceutical sector is the tax called *Imposto sobre Circulação de Mercadorias e Serviços* (ICMS), which is the responsibility of each state and is part of the maximum sale price to the consumer. The values paid by the pharmaceutical sector to the ICMS tax reach 18%, while the values paid for cars and food, for example, do not exceed 12% and 8%, respectively [18].

An analysis of different types of medicines suggests low availability of antidepressants, anti-epileptics and anxiolytics in the public system. This could be explained by the way these medicines are dispensed. Because their dispensing is tightly regulated, they are available in specific facilities in some cities. If we consider only the facilities which are able to distribute these medicines, availability ranges from 75 to 100%.

Caution should be exercised when extrapolating our data to the national level because there may be regional differences in public and private sector availability of medicines across the different regions of the country. Such differences may reflect different priorities in health care given the different profiles of morbidity outside the study region. Still, our results compare well with other studies conducted in Brazil. There may be differences in prices between this region and other regions in the country, partly because of differences in sales taxes, but these are marginal and are unlikely to affect our comparisons with other national-level evidence. Because the proportion of informal work varies considerably across regions of the country, care should be exercised when extrapolating our affordability findings to the national level.

Finally, there are inherent limitations relating to the WHO/HAI methodology. One of them refers to the availability measure, considering that it is not related to stock levels and might not indicate average availability. However, the data provide an estimate of the overall situation of a given place. This and other limitations of the WHO/HAI methodology have been discussed elsewhere [17].

## Conclusions

High prices, poor availability in public sector facilities and low affordability suggest a number of policy implications for the Brazilian government. First, it needs to maintain its commitment of providing a list of essential medicines free of charge at public facilities, aiming to fulfill the target of 100% availability for this list of medicines. Second, the participation of generic products in the market needs to be increased and their prices reduced further, through better tendering processes, so that generics become cheaper than similar products. Although generics and similar medicines are alternatives to originator brands, they are also 8.6 - 11.3 more expensive than international reference prices. Therefore, an overall reduction in medicine prices should be a key priority. In order to achieve this, reductions in taxes and duties on medicines, and margin regulations in the supply chain could be considered. Policy measures used in developed country settings and evidence on their performance could be used in the Brazilian context [14]. Third, the routines of acquisition, stock and distribution of medicines in the public sector need to be re-evaluated, ensuring adequate and timely distribution of essential medicines. The use of information and communication technologies should be prioritized. Fourth, the expansion of the list of medicines regulated by the government is one approach to include all medicines provided by SUS. This expansion would lead to the purchase of medicines at lower prices by the government. Fifth, if the government continues to be unable to provide for free all essential medicines needed by the population, it is necessary to expand the popular pharmacy programme, by increasing the number of facilities and the number of medicines available. Alongside that, the quantity and quality of information available to patients about prices should also improve. Finally, the private market requires tighter regulation, so that only OTC medicines are dispensed without a prescription.

## Endnotes

^a^Key medicines are those suitable and used for international comparisons (global and regional list) or commonly used therapeutic alternatives to those which are part of the global and regional list. ^b^Ordinary people are defined as the lowest-paid unskilled government worker. ^c^All collected data (price and availability) were entered in the workbook, consolidated and summarized. Data were entered twice in order to avoid entry errors. Based on the unique feature of the Brazilian medicines market, comprising originators, generics and similar medicines, three workbooks were analyzed: originator brands vs. generics, originator brands vs. similar medicines, and generics vs. similar medicines.

## Competing interest

The authors declare that they have no competing interest.

## Author’s contributions

AB coordinated the conception, design, acquisition of data, analysis and interpretation of data and was the main investigator involved in drafting the manuscript. APH participated in the acquisition of data and carried out all analysis. ALC and NULT made substantial contributions to conception and design and participated in the acquisition of data. PK revised the manuscript critically for important intellectual content. All authors read and approved the final version of the manuscript to be published.

## Supplementary Material

Additional file 1 ** Table S1.**List of the medicines investigated (N=50) according to therapeutic classes, methodology list belonged to, presence in the Brazilian list of essential medicines (RENAME) and in the Popular Pharmacy Programme, and mean % availability in the public and private sector of the three types of medicines (originator brand, similar medicines and generics). Sample of six cities from the Rio Grande do Sul state, Brazil, 2008-9. Click here for file
