# Supplementary material for: Is the Brazilian pharmaceutical policy ensuring population access to essential medicines?
Source: Global Health. 2012 Mar 21;8:6. doi: 10.1186/1744-8603-8-6 (PMC3511298; doi:10.1186/1744-8603-8-6)
Supplement: Additional file 1 — Table S1.List of the medicines investigated (N=50) according to therapeutic classes, methodology list belonged to, presence in the Brazilian list of essential medicines (RENAME) and in the Popular Pharmacy Programme, and mean % availability in the public and private sector of the three types of medicines (originator brand, similar medicines and generics). Sample of six cities from the Rio Grande do Sul state, Brazil, 2008-9. [file 1744-8603-8-6-S1.doc]

| **Table 1.** List of the medicines investigated (N=50) according to therapeutic classes, methodology list belonged to, presence in the Brazilian list of essential medicines (RENAME) and in the Popular Pharmacy Programme, and mean % availability in the public and private sector of the three types of medicines (originator brand, similar medicines and generics). Sample of six cities from the Rio Grande do Sul state, Brazil, 2008-9.   | **Therapeutic class** | **Medicine** | | | **Methodology list** | | | **RENAME**  **2008** | | **Popular pharmacy** | | | **Mean % availability** | | | | | | | | | | | | | | | | | | | --- | --- | --- | --- | --- | --- | --- | --- | --- | --- | --- | --- | --- | --- | --- | --- | --- | --- | --- | --- | --- | --- | --- | --- | --- | --- | --- | --- | --- | --- | | **Public^1^** | | | | | **Private** | | | | | | | | | | | | | | **N^2^** | **Generic** | | **Similar** | | |  | **N^3^** | | **Generic** | | **N^4^** | | **Similar** | | | | **Originator brand** | | Analgesic | Paracetamol 500 mg tab | | | Supplementary | | | Yes | | Yes | | | 5 | 77.8 | | 11.1 | | |  | 12 | | 46.7 | | 14 | | 60.0 | | | | 60.0 | | Anthelmintic | Mebendazole 20 mg/ml susp | | | Supplementary | | | No | | Yes | | | 6 | 9.1 | | 86.4 | | |  | 16 | | 83.3 | | 30 | | 50.0 | | | | 83.3 | | Antidepressant | Amitriptyline 25 mg tab^5^ | | | Global | | | Yes | | Yes | | | 6 | 4.5 | | 27.3 | | |  | 10 | | 86.7 | | 6 | | 53.3 | | | | 70.0 | | Fluoxetine 20 mg cap^5^ | | | Regional | | | Yes | | No | | | 1 | 0 | | 25.0 | | |  | 11 | | 93.3 | | 11 | | 70.0 | | | | 26.7 | | Antidiabetis | Glibenclamide 5 mg tab | | | Global | | | Yes | | Yes | | | 6 | 0 | | 100.0 | | |  | 7 | | 73.3 | | 15 | | 70.0 | | | | 83.3 | | Metformin 850 mg tab | | | Regional | | | Yes | | Yes | | | 6 | 22.7 | | 77.3 | | |  | 17 | | 90.0 | | 14 | | 56.7 | | | | 90.0 | | Antiepileptic | Carbamazepine 200 mg tab^5^ | | | Supplementary | | | Yes | | Yes | | | 6 | 4.5 | | 27.3 | | |  | 11 | | 86.7 | | 6 | | 13.3 | | | | 66.7 | | Clonazepam 2mg tab^5^ | | | Regional | | | Yes | | No | | | 2 | 0 | | 25.0 | | |  | 5 | | 43.3 | | 5 | | 16.7 | | | | 93.3 | | Phenytoin 100 mg tab^5^ | | | Regional | | | Yes | | Yes | | | 6 | 22.7 | | 9.1 | | |  | 3 | | 60.0 | | 3 | | 3.3 | | | | 93.3 | | Antifungic | Miconazole 20 mg/g cr | | | Supplementary | | | Yes | | No | | | 5 | 56.6 | | 16.7 | | |  | 9 | | 83.3 | | 10 | | 16.7 | | | | 33.3 | | Nystatin 100,000 UI/ml susp | | | Supplementary | | | Yes | | Yes | | | 4 | 57.5 | | 0 | | |  | 9 | | 90.0 | | 16 | | 30.0 | | | | 63.3 | | Antihypertensive | Amlodipine 5 mg tab | | | Regional | Yes | | | | No | | | 0 | - | | - | | |  | 16 | | 76.7 | | 14 | | 46.7 | | | | 36.7 | | Atenolol 50 mg tab | | | Global | | Yes | | | No | | | 0 | - | | - | | |  | 17 | | 90.0 | | 13 | | 76.7 | | | | 76.7 | | Captopril 25 mg tab | | | Global | | Yes | | | Yes | | | 6 | 18.2 | | 81.8 | | |  | 16 | | 86.7 | | 35 | | 76.7 | | | | 36.7 | | Enalapril 10 mg tab | | | Regional | | Yes | | | Yes | | | 3 | 0 | | 66.6 | | |  | 15 | | 86.7 | | 29 | | 93.3 | | | | 60.0 | | Methyldopa 250 mg tab | | | Supplementary | | Yes | | | Yes | | | 5 | 0 | | 83.3 | | |  | 4 | | 70.0 | | 16 | | 40.0 | | | | 70.0 | | Nifedipine 10mg tab | | | Supplementary | | Yes | | | No | | | 5 | 0 | | 59.0 | | |  | 0 | | 0 | | 5 | | 36.7 | | | | 66.7 | | Propranolol 40 mg tab | | | Supplementary | | Yes | | | Yes | | | 6 | 0 | | 100.0 | | |  | 8 | | 80.0 | | 20 | | 63.3 | | | | 73.3 | |  | Verapamil 80 mg tab | | | Supplementary | | Yes | | | Yes | | | 4 | 12.5 | | 71.3 | | |  | 8 | | 90.0 | | 6 | | 26.7 | | | | 70.0 | | Antiinfective | Amoxicillin 500 mg tab | | | Global | | Yes | | | Yes | | | 6 | 31.8 | | 68.2 | | |  | 23 | | 100.0 | | 28 | | 60.0 | | | | 56.7 | | Amoxicillin 50 mg/ml susp | | | Regional | | Yes | | | Yes | | | 6 | 0 | | 90.9 | | |  | 14 | | 93.3 | | 12 | | 53.3 | | | | 63.3 | | Azithromycin 500 mg tab | | | Regional | | Yes | | | Yes | | | 1 | 0 | | 25.0 | | |  | 10 | | 73.3 | | 19 | | 86.7 | | | | 26.7 | | Benzylpenicillin-benzathin 1200000 U.I./vial inj | | | Supplementary | | Yes | | | Yes | | | 6 | 0 | | 77.3 | | |  | 0 | | 0 | | 2 | | 0 | | | | 90.0 | | Cefalexin 500 mg tab | | | Supplementary | | Yes | | | Yes | | | 5 | 33.3 | | 22.2 | | |  | 13 | | 86.7 | | 16 | | 16.7 | | | | 60.0 | | ...continuation of table 1. | | | | | | | | | | | | | | | | | | | | | | | | | | | | | | | **Therapeutic class** | | **Medicine** | **Methodology list** | | | **RENAME**  **2008** | | **Popular pharmacy** | | **Mean % availability** | | | | | | | | | | | | | | | | | | | | | **Public^1^** | | | | | | | **Private** | | | | | | | | | |  | | | | **N^2^** | **Generic** | | | **Similar** | |  | **N^3^** | | | **Generic** | | **N^4^** | | **Similar** | | | **Originator brand** | | | | Antiinfective | | Cefalexina 50 mg/ml susp | Supplementary | | | Yes | | Yes | | 5 | 22.2 | | | 44.4 | |  | 7 | | | 83.3 | | 11 | | 30.0 | | | 40.0 | | | | Ceftriaxone 1g/vial inj | Global | | | Yes | | No | | 0 | - | | | - | |  | 9 | | | 66.7 | | 3 | | 23.3 | | | 20.0 | | | | Ciprofloxacin 500mg tab | Global | | | Yes | | Yes | | 1 | 0 | | | 25.0 | |  | 15 | | | 80.0 | | 21 | | 66.7 | | | 33.3 | | | | Co-trimoxazole 80+400mg tab | Supplementary | | | Yes | | Yes | | 6 | 9.1 | | | 77.3 | |  | 1 | | | 26.7 | | 28 | | 73.3 | | | 86.7 | | | | Co-trimoxazole 8+40mg/ml susp | Global | | | Yes | | Yes | | 6 | 0 | | | 72.7 | |  | 3 | | | 66.7 | | 28 | | 53.3 | | | 93.3 | | | | Clotrimazole 10 mg/g cr | Regional | | | No | | No | | 0 | - | | | - | |  | 12 | | | 66.7 | | 18 | | 43.3 | | | 96.7 | | | | Doxycycline 100 mg tab | Supplementary | | | Yes | | Yes | | 3 | 0 | | | 91.6 | |  | 1 | | | 50.0 | | 7 | | 36.7 | | | 53.3 | | | | Erythromycin 500 mg tab | Supplementary | | | Yes | | Yes | | 6 | 0 | | | 86.4 | |  | 0 | | | 0 | | 10 | | 53.3 | | | 63.3 | | | | Erythromycin 50 mg/ml susp | Supplementary | | | Yes | | Yes | | 6 | 0 | | | 77.3 | |  | 0 | | | 0 | | 7 | | 30.0 | | | 53.3 | | | | Metronidazole 250 mg tab | Supplementary | | | Yes | | Yes | | 5 | 38.9 | | | 27.8 | |  | 3 | | | 63.3 | | 16 | | 20.0 | | | 73.3 | | | | Metronidazole 400 mg tab | Regional | | | Yes | | No | | 2 | 37.5 | | | 25.0 | |  | 2 | | | 73.3 | | 4 | | 33.3 | | | 86.7 | | | | Antiinflammatory | | Bec lometasone 250 mcg/dose inhaler | Regional | | | Yes | | No | | 3 | 0 | | | 0 | |  | 0 | | | 0 | | 1 | | 10.0 | | | 46.7 | | | | Dic lofenac sodium 50 mg tab | Global | | | No | | No | | 1 | 0 | | | 75.0 | |  | 9 | | | 76.7 | | 36 | | 93.3 | | | 90.0 | | | | Ibuprofen 400 mg cap | Regional | | | No | | No | | 0 | - | | | - | |  | 0 | | | 0 | | 0 | | 0 | | | 0 | | | | Prednisone 5 mg tab | Supplementary | | | Yes | | Yes | | 5 | 11.1 | | | 88.9 | |  | 10 | | | 90.0 | | 10 | | 60.0 | | | 93.3 | | | | Antithrombotic agent | | Acetylsalicylic acid 100mg tab | Supplementary | | | Yes | | Yes | | 6 | 18.2 | | | 86.4 | |  | 5 | | | 76.7 | | 8 | | 86.7 | | | 86.7 | | | | Anxiolytic | | Diazepam 5 mg tab^5^ | Global | | | Yes | | Yes | | 2 | 0 | | | 25.0 | |  | 3 | | | 70.0 | | 7 | | 46.7 | | | 70.0 | | | | Cardiac therapy | | Digoxin 0,25 mg tab | Supplementary | | | Yes | | Yes | | 6 | 40.9 | | | 63.6 | |  | 3 | | | 73.3 | | 9 | | 46.7 | | | 73.3 | | | | Diuretic | | Furosemide 40 mg tab | Regional | | | Yes | | Yes | | 6 | 0 | | | 100.0 | |  | 6 | | | 70.0 | | 17 | | 76.7 | | | 93.3 | | | | Hydrochlorothiazide 25 mg tab | Regional | | | Yes | | Yes | | 6 | 9.1 | | | 95.5 | |  | 5 | | | 56.7 | | 5 | | 83.3 | | | 90.0 | | | | Drug for acid related disorder | | Omeprazole 20 mg cap | Global | | | Yes | | Yes | | 4 | 0 | | | 82.5 | |  | 10 | | | 86.7 | | 37 | | 90.0 | | | 46.7 | | | | Ranitidine 150 mg tab | Regional | | | Yes | | Yes | | 1 | 0 | | | 100.0 | |  | 12 | | | 76.7 | | 27 | | 43.3 | | | 43.3 | | | | Drug for obstructive airway disease | | Salbutamol 100mcg/dose inhaler | Global | | | Yes | | No | | 2 | 0 | | | 75.0 | |  | 0 | | | 0 | | 3 | | 63.3 | | | 86.7 | | | |  | | Salbutamol xar 2mg/5ml syrup | Supplementary | | | No | | Yes | | 6 | 45.5 | | | 31.8 | |  | 11 | | | 80.0 | | 21 | | 10.0 | | | 73.3 | | | |  | |  |  | | |  | |  | |  |  | | |  | |  |  | | |  | |  | |  | | |  | | | | [Lipide modifying agent](http://www.whocc.no/atc_ddd_index/?code=C10) | | Atorvastatin 10 mg tab | Regional | | | No | | No | | 0 | - | | | - | |  | 0 | | | 0 | | 1 | | 20.0 | | | 53.3 | | | |  | | Simvastatin 20 mg tab | Global | | | No | | Yes | | 0 | - | | | - | |  | 7 | | | 70.0 | | 15 | | 93.3 | | | 33.3 | | | | ^1^ No originator brand was located in the public sector ^2^ Out of the six cities studied, in how many the medicine was part of the list of essential medicines ^3^ Number of generics available in the market ^4^ Number of similar medicines available in the market ^5^ Medicines subject to especial control. In some cities, they are distributed in specific pharmacies only | | | | | | | | | | | | | | | | | | | | | | | | | |  | |  | | |  | |  | | |  |  |  |  |  |  |  |  |  |  |  |  |  |
| --- | --- | --- | --- | --- | --- | --- | --- | --- | --- | --- | --- | --- | --- | --- | --- | --- | --- | --- | --- | --- | --- | --- | --- | --- | --- | --- | --- | --- | --- | --- | --- | --- | --- | --- | --- | --- | --- | --- | --- | --- | --- | --- | --- | --- | --- | --- | --- | --- | --- | --- | --- | --- | --- | --- | --- | --- | --- | --- | --- | --- | --- | --- | --- | --- | --- | --- | --- | --- | --- | --- | --- | --- | --- | --- | --- | --- | --- | --- | --- | --- | --- | --- | --- | --- | --- | --- | --- | --- | --- | --- | --- | --- | --- | --- | --- | --- | --- | --- | --- | --- | --- | --- | --- | --- | --- | --- | --- | --- | --- | --- | --- | --- | --- | --- | --- | --- | --- | --- | --- | --- | --- | --- | --- | --- | --- | --- | --- | --- | --- | --- | --- | --- | --- | --- | --- | --- | --- | --- | --- | --- | --- | --- | --- | --- | --- | --- | --- | --- | --- | --- | --- | --- | --- | --- | --- | --- | --- | --- | --- | --- | --- | --- | --- | --- | --- | --- | --- | --- | --- | --- | --- | --- | --- | --- | --- | --- | --- | --- | --- | --- | --- | --- | --- | --- | --- | --- | --- | --- | --- | --- | --- | --- | --- | --- | --- | --- | --- | --- | --- | --- | --- | --- | --- | --- | --- | --- | --- | --- | --- | --- | --- | --- | --- | --- | --- | --- | --- | --- | --- | --- | --- | --- | --- | --- | --- | --- | --- | --- | --- | --- | --- | --- | --- | --- | --- | --- | --- | --- | --- | --- | --- | --- | --- | --- | --- | --- | --- | --- | --- | --- | --- | --- | --- | --- | --- | --- | --- | --- | --- | --- | --- | --- | --- | --- | --- | --- | --- | --- | --- | --- | --- | --- | --- | --- | --- | --- | --- | --- | --- | --- | --- | --- | --- | --- | --- | --- | --- | --- | --- | --- | --- | --- | --- | --- | --- | --- | --- | --- | --- | --- | --- | --- | --- | --- | --- | --- | --- | --- | --- | --- | --- | --- | --- | --- | --- | --- | --- | --- | --- | --- | --- | --- | --- | --- | --- | --- | --- | --- | --- | --- | --- | --- | --- | --- | --- | --- | --- | --- | --- | --- | --- | --- | --- | --- | --- | --- | --- | --- | --- | --- | --- | --- | --- | --- | --- | --- | --- | --- | --- | --- | --- | --- | --- | --- | --- | --- | --- | --- | --- | --- | --- | --- | --- | --- | --- | --- | --- | --- | --- | --- | --- | --- | --- | --- | --- | --- | --- | --- | --- | --- | --- | --- | --- | --- | --- | --- | --- | --- | --- | --- | --- | --- | --- | --- | --- | --- | --- | --- | --- | --- | --- | --- | --- | --- | --- | --- | --- | --- | --- | --- | --- | --- | --- | --- | --- | --- | --- | --- | --- | --- | --- | --- | --- | --- | --- | --- | --- | --- | --- | --- | --- | --- | --- | --- | --- | --- | --- | --- | --- | --- | --- | --- | --- | --- | --- | --- | --- | --- | --- | --- | --- | --- | --- | --- | --- | --- | --- | --- | --- | --- | --- | --- | --- | --- | --- | --- | --- | --- | --- | --- | --- | --- | --- | --- | --- | --- | --- | --- | --- | --- | --- | --- | --- | --- | --- | --- | --- | --- | --- | --- | --- | --- | --- | --- | --- | --- | --- | --- | --- | --- | --- | --- | --- | --- | --- | --- | --- | --- | --- | --- | --- | --- | --- | --- | --- | --- | --- | --- | --- | --- | --- | --- | --- | --- | --- | --- | --- | --- | --- | --- | --- | --- | --- | --- | --- | --- | --- | --- | --- | --- | --- | --- | --- | --- | --- | --- | --- | --- | --- | --- | --- | --- | --- | --- | --- | --- | --- | --- | --- | --- | --- | --- | --- | --- | --- | --- | --- | --- | --- | --- | --- | --- | --- | --- | --- | --- | --- | --- | --- | --- | --- | --- | --- | --- | --- | --- | --- | --- | --- | --- | --- | --- | --- | --- | --- | --- | --- | --- | --- | --- | --- | --- | --- | --- | --- | --- | --- | --- | --- | --- | --- | --- | --- | --- | --- | --- | --- | --- | --- | --- | --- | --- | --- | --- | --- | --- | --- | --- | --- | --- | --- | --- | --- | --- | --- | --- | --- | --- | --- | --- | --- | --- | --- | --- | --- | --- | --- | --- | --- | --- | --- | --- | --- | --- | --- | --- | --- | --- | --- | --- | --- | --- | --- | --- | --- | --- | --- | --- | --- | --- | --- | --- | --- | --- | --- | --- | --- | --- | --- | --- | --- | --- | --- | --- | --- | --- | --- | --- | --- | --- | --- | --- | --- | --- | --- | --- | --- | --- | --- | --- | --- | --- | --- | --- | --- | --- | --- | --- | --- | --- | --- | --- | --- | --- | --- | --- | --- | --- | --- | --- | --- | --- | --- | --- | --- | --- | --- | --- | --- | --- | --- | --- | --- | --- | --- | --- | --- | --- | --- | --- | --- | --- | --- | --- | --- | --- | --- | --- | --- | --- | --- | --- | --- | --- | --- | --- | --- | --- | --- | --- | --- | --- | --- | --- | --- | --- | --- | --- | --- | --- | --- | --- | --- | --- | --- | --- | --- | --- | --- | --- | --- | --- | --- | --- | --- | --- | --- | --- | --- | --- | --- | --- | --- | --- | --- | --- | --- | --- | --- | --- | --- | --- | --- | --- | --- | --- | --- | --- | --- | --- | --- | --- | --- | --- | --- | --- | --- | --- | --- | --- | --- | --- | --- | --- | --- | --- | --- | --- | --- | --- | --- | --- | --- | --- | --- | --- | --- | --- | --- | --- | --- | --- | --- | --- | --- | --- | --- | --- | --- | --- | --- | --- | --- | --- | --- | --- | --- | --- | --- | --- | --- | --- | --- | --- | --- | --- | --- | --- | --- | --- | --- | --- | --- | --- | --- | --- | --- | --- | --- | --- | --- | --- | --- | --- | --- | --- | --- | --- | --- | --- | --- | --- | --- | --- | --- | --- | --- | --- | --- | --- | --- | --- | --- | --- | --- | --- | --- | --- | --- | --- | --- | --- | --- | --- | --- | --- | --- | --- | --- | --- | --- | --- | --- | --- | --- | --- | --- | --- | --- | --- | --- | --- | --- | --- | --- | --- | --- | --- | --- | --- | --- | --- | --- | --- | --- | --- | --- | --- | --- | --- | --- | --- | --- | --- | --- | --- | --- | --- | --- | --- | --- | --- | --- | --- | --- | --- | --- | --- | --- | --- | --- | --- | --- | --- | --- | --- | --- | --- | --- | --- | --- | --- | --- | --- | --- | --- | --- | --- | --- | --- | --- | --- | --- | --- | --- | --- | --- | --- | --- | --- | --- | --- | --- | --- | --- | --- | --- | --- | --- | --- | --- | --- | --- | --- | --- | --- | --- | --- | --- | --- | --- | --- | --- | --- | --- | --- | --- | --- | --- | --- | --- | --- | --- | --- | --- | --- | --- | --- | --- | --- | --- | --- | --- | --- | --- | --- | --- | --- | --- | --- | --- | --- | --- | --- | --- | --- | --- | --- | --- | --- | --- | --- | --- | --- | --- | --- | --- | --- | --- | --- | --- | --- | --- | --- | --- | --- | --- | --- | --- | --- | --- | --- | --- | --- | --- | --- | --- | --- | --- | --- | --- | --- | --- | --- | --- | --- | --- | --- | --- | --- | --- | --- | --- | --- | --- | --- | --- | --- | --- | --- | --- | --- | --- | --- | --- | --- | --- | --- | --- | --- | --- | --- | --- | --- | --- | --- | --- | --- | --- | --- | --- | --- | --- | --- | --- | --- | --- | --- | --- | --- | --- | --- | --- | --- | --- | --- | --- | --- | --- | --- | --- | --- | --- | --- | --- | --- | --- | --- | --- | --- | --- | --- | --- | --- | --- | --- | --- | --- | --- | --- | --- | --- | --- | --- | --- | --- | --- | --- | --- | --- | --- | --- | --- | --- | --- | --- | --- | --- | --- | --- | --- | --- | --- | --- | --- | --- | --- | --- | --- | --- | --- | --- | --- | --- | --- | --- | --- | --- | --- | --- | --- | --- | --- | --- | --- | --- | --- | --- | --- | --- | --- | --- | --- | --- | --- | --- | --- | --- | --- | --- | --- | --- | --- | --- | --- | --- | --- | --- | --- | --- | --- | --- | --- | --- | --- | --- | --- | --- | --- | --- | --- | --- | --- | --- | --- | --- | --- | --- | --- | --- | --- | --- | --- | --- | --- | --- | --- | --- | --- | --- | --- | --- | --- | --- | --- | --- | --- | --- | --- | --- | --- | --- | --- | --- | --- | --- | --- | --- | --- | --- | --- | --- | --- | --- | --- | --- | --- | --- | --- | --- | --- | --- | --- | --- | --- | --- | --- | --- | --- | --- | --- | --- | --- | --- | --- | --- | --- | --- | --- | --- | --- | --- | --- | --- | --- | --- | --- | --- | --- | --- | --- | --- | --- | --- | --- | --- | --- | --- | --- | --- | --- | --- | --- | --- | --- | --- | --- | --- | --- | --- | --- | --- | --- | --- | --- | --- | --- | --- | --- | --- | --- | --- | --- | --- | --- | --- | --- | --- | --- | --- | --- | --- | --- | --- | --- | --- | --- | --- | --- | --- | --- | --- | --- | --- | --- | --- | --- | --- | --- | --- | --- | --- | --- | --- | --- | --- | --- | --- | --- | --- | --- | --- | --- | --- | --- | --- | --- | --- | --- | --- | --- | --- | --- | --- | --- | --- | --- | --- | --- | --- | --- | --- | --- | --- | --- | --- | --- | --- | --- | --- | --- | --- | --- | --- | --- | --- | --- | --- | --- | --- | --- | --- | --- | --- | --- | --- | --- | --- | --- | --- | --- | --- | --- | --- | --- | --- | --- | --- | --- | --- | --- | --- | --- | --- | --- | --- | --- | --- | --- | --- | --- | --- | --- | --- | --- | --- | --- | --- | --- | --- | --- | --- | --- | --- | --- | --- | --- | --- | --- | --- | --- | --- | --- | --- | --- | --- | --- | --- | --- | --- | --- | --- | --- | --- | --- | --- | --- | --- | --- | --- | --- | --- | --- | --- | --- | --- | --- | --- | --- | --- | --- | --- | --- | --- | --- | --- | --- | --- | --- | --- | --- | --- | --- | --- | --- | --- | --- | --- | --- | --- | --- | --- | --- | --- | --- | --- | --- | --- | --- | --- | --- | --- | --- | --- | --- | --- | --- | --- | --- | --- | --- | --- | --- | --- | --- | --- | --- | --- | --- | --- | --- | --- | --- | --- | --- | --- | --- | --- | --- | --- | --- | --- | --- | --- | --- | --- | --- | --- | --- | --- | --- | --- | --- | --- | --- | --- | --- | --- | --- | --- | --- | --- | --- | --- | --- | --- | --- | --- | --- | --- | --- | --- | --- | --- | --- | --- | --- | --- | --- | --- | --- | --- | --- | --- | --- | --- | --- | --- | --- | --- | --- | --- | --- | --- | --- | --- | --- | --- | --- | --- | --- | --- | --- | --- | --- | --- | --- | --- | --- | --- | --- | --- | --- | --- | --- | --- | --- | --- | --- | --- | --- | --- | --- | --- | --- | --- | --- | --- | --- | --- | --- | --- | --- | --- | --- | --- | --- | --- | --- | --- | --- | --- | --- | --- | --- | --- | --- | --- |
